# Estrogen signaling as a bridge between the nucleus and mitochondria in cardiovascular diseases

**DOI:** 10.3389/fcell.2022.968373

**Published:** 2022-09-14

**Authors:** Emanuel Guajardo-Correa, Juan Francisco Silva-Agüero, Ximena Calle, Mario Chiong, Mauricio Henríquez, Gerardo García-Rivas, Mauricio Latorre, Valentina Parra

**Affiliations:** ^1^ Advanced Center of Chronic Diseases (ACCDiS), Facultad de Ciencias Químicas y Farmacéuticas y Facultad de Medicina, Universidad de Chile, Santiago, Chile; ^2^ Departamento de Bioquímica y Biología Molecular, Facultad de Ciencias Químicas y Farmacéuticas, Universidad de Chile, Santiago, Chile; ^3^ Escuela de Química y Farmacia, Facultad de Medicina, Universidad Andres Bello, Santiago, Chile; ^4^ Center of Applied Nanoscience (CANS), Facultad de Ciencias Exactas, Universidad Andres Bello, Santiago, Chile; ^5^ Programa de Fisiología y Biofísica, Instituto de Ciencias Biomédicas, Facultad de Medicina, Universidad de Chile, Santiago, Chile; ^6^ Red para el Estudio de Enfermedades Cardiopulmonares de Alta Letalidad (REECPAL), Universidad de Chile, Santiago, Chile; ^7^ Tecnológico de Monterrey, Escuela de Medicina y Ciencias de la Salud, Monterrey, Nuevo León, Mexico; ^8^ Tecnológico de Monterrey, The Institute for Obesity Research, Hospital Zambrano Hellion, San Pedro Garza Garcia, Nuevo León, Mexico; ^9^ Laboratorio de Bioingeniería, Instituto de Ciencias de la Ingeniería, Universidad de O’Higgins, Rancagua, Chile; ^10^ Laboratorio de Bioinformática y Expresión Génica, INTA, Universidad de Chile, Santiago, Chile

**Keywords:** estrogens, nucleus, mitochondria, cardiovascular diseases, cardiac hypertrophy, heart failure

## Abstract

Cardiovascular diseases (CVDs) are the leading cause of morbidity and mortality worldwide. Epidemiological studies indicate that pre-menopausal women are more protected against the development of CVDs compared to men of the same age. This effect is attributed to the action/effects of sex steroid hormones on the cardiovascular system. In this context, estrogen modulates cardiovascular function in physiological and pathological conditions, being one of the main physiological cardioprotective agents. Here we describe the common pathways and mechanisms by which estrogens modulate the retrograde and anterograde communication between the nucleus and mitochondria, highlighting the role of genomic and non-genomic pathways mediated by estrogen receptors. Additionally, we discuss the presumable role of bromodomain-containing protein 4 (BRD4) in enhancing mitochondrial biogenesis and function in different CVD models and how this protein could act as a master regulator of estrogen protective activity. Altogether, this review focuses on estrogenic control in gene expression and molecular pathways, how this activity governs nucleus-mitochondria communication, and its projection for a future generation of strategies in CVDs treatment.

## 1 Introduction

Cardiovascular diseases (CVDs) are the leading cause of morbidity and mortality worldwide (Virani et al., 2021). The most common CVDs are stroke, heart failure, coronary artery diseases, hypertension, heart arrhythmia, peripheral artery disease, and atherosclerosis, which are characterized by heart and/or blood vessel dysfunction (Virani et al., 2021). High blood pressure, high blood glucose, smoking, obesity, lack of exercise, alcohol consumption, and dyslipidemia are the main risk factors for the development of CVDs and they can be modified by gender, race and ethnicity (Hu et al., 2017; Virani et al., 2021). Interestingly, when these risk factors are reduced, the CVDs mortality rates decrease by 50%, as well as the use of other preventive therapies (Ford et al., 2007).

Currently, ageing is an inevitable determinant in CVDs, leading to decreased mitochondrial functions, excessive production of reactive oxygen species (ROS), and altered calcium (Ca^2+^) levels, which are important determinants for the progressive damage in several physiological processes and that increase the incidence of hypertension, atherosclerosis and cerebrovascular accidents. Moreover, there is evidence that shows a strong relationship between the nucleus and mitochondria function in controlling the expression of key genes involved in CVDs (North and Sinclair, 2012; Almeida et al., 2017).

Several studies have reported the different rates of CVDs among men and women (O’Neil et al., 2018). Epidemiological studies have indicated that pre-menopausal women are more protected against the development of CVDs compared to men of the same age (Mosca et al., 2011). This cardioprotective effect is attributed to the sex hormones levels in this group (Yang and Reckelhoff, 2011). In recent years, sufficient evidence has supported the idea that the differences in vascular biology between men and women are related to the cardiovascular and metabolic action/effects of sex steroid hormones (Vitale et al., 2010). Estrogen modulates cardiovascular physiology and function in physiological and pathological conditions, being one of the main physiological cardioprotective agents (Ford et al., 2007). Thus, unveiling the action mechanism and role of estrogen in the integration of organelle function will help elucidate new therapeutic targets to fight CVDs and propose that the difference in its levels may play a key role in cardiovascular pathophysiology (Vitale et al., 2010). Sex steroid hormones exert both direct and indirect effects on cardiovascular functions due to their metabolic and vasoactive properties, which are mediated by genomic and non-genomic mechanisms (Tian and Meng, 2019). All these actions will be discussed in detail below. Thus, this article will review the effects of estrogen at the cardiovascular level and its role in the coordination between mitochondria and nucleus functioning in the context of CVDs.

## 2 Estrogens and cardiovascular diseases

Estrogens exert essential effects on the cardiovascular system, and their actions depend on factors such as dose/concentration, target tissue, gender, estrogen receptor (ER) subtype expressed in the tissue, and the developmental period of age where the measures were developed (Iorga et al., 2017). Moreover, as estrogens can be generated and secreted by different types and tissues, their effects on proliferation and mitochondrial bioenergetics are common between different cell types, no matter the distance to the target tissue or if the secretion is considered endocrine, autocrine, or paracrine (Lang, 2004; Deroo and Korach, 2006; Bustamante-Barrientos et al., 2021). The most common and predominant form of circulating estrogen, as well as the primary female sex hormone, is 17 β-estradiol (E2) (Murphy, 2011). In premenopausal women, E2 is synthesized and secreted predominantly by the ovaries and other tissue types, such as adipose, brain, and bone tissues, as well as in the vascular endothelium and aortic smooth muscle cells (Bayard et al., 2007). Postmenopausal women are at higher risk of CVDs than premenopausal women and men of the same age. Estrogen exerts several beneficial effects on vascular function, such as improving the lipid profile, increasing the mitochondrial function, reducing atherosclerosis and fibrosis, decreasing oxidative stress, attenuating cardiac hypertrophy (CH), and stimulating angiogenesis and vasodilatation (Mendelsohn, 2002; Murphy, 2011; Iorga et al., 2017). We will discuss most of these effects in detail in the next subsections.

### 2.1 Potential estrogen effects in cardiovascular diseases

Several studies have shown that estrogen can delay the development of CH. Thus, in a model of ovariectomized (OVX) mice subjected to transverse aortic constriction (TAC), E2 prevented HC due to pressure overload, reducing CH by 31% through decreased p38-mitogen-activated protein kinase (MAPK) phosphorylation. Thus, E2 has a direct beneficial effect on the heart and could therefore, reduce the prevalence of CH in postmenopausal women (Eickels et al., 2001).

In parallel, estrogen induces the expression of endothelial nitric oxide synthase (eNOS) and inducible nitric oxide synthase (iNOS) in neonatal and adult cardiomyocytes both *in vitro* and *in vivo*, and is able to modulate nitric oxide synthase (NOS) expression and nitric oxide (NO) formation in the myocardium, protecting it against inflammation (Nuedling et al., 1999). The induction of neoangiogenesis through E2 therapy depends on the activation of eNOS, since mice without eNOS do not exhibit proangiogenic effects after E2 therapy (Cai and Harrison, 2000; Iorga et al., 2017). NO can induce post-translational protein modifications, such as protein S-nitrosylation of cysteine, which may exert anti-inflammatory effects. Estrogen protects hearts against ischemia/reperfusion (I/R) injury by activating the estrogen receptor beta (ERβ), NO/NOS signaling and S-nitrosylation in the vascular endothelia (Lin et al., 2009; Chakrabarti et al., 2010). In a study performed in female mice with angiotensin II-induced hypertension, E2 also showed acute and chronic vasodilation activity, decreasing arterial hypertension through a NO and estrogen receptor alpha (ERα)-mediated pathway (Guivarc’h et al., 2018). Additionally, E2 can also attenuate ERβ mediated vasoconstriction in mice through iNOS expression (Zhu et al., 2002). Therefore, E2 plays a role in modulating vasorelaxation (White et al., 2005), vasoconstriction inhibition (Gallagher et al., 1999) and endothelial function through eNOS-dependent mechanisms, contributing to the direct cardioprotective effect of E2 in reducing CH and improving cardiac function (Iorga et al., 2017).

Another CVD where estrogen intervenes is in pulmonary arterial hypertension (PH). Females developed a less severe PH, compared to males. In a study with E2 pretreatment, the severity of PH was reduced in both female and male rats (Farhat et al., 1993), whereas another study demonstrated that estrogen receptors (ER) are involved in the protective effect of E2 in PH by using specific ERα agonists in rats (Frump et al., 2015). In contrast, other studies have indicated that ERβ is a cardiopulmonary protective receptor whose activation elicits vasoconstrictive, antiproliferative right ventricular hypertrophy and antifibrotic response, suggesting that both receptors are involved in the process, as well as the G protein-coupled estrogen receptor (GPER), which also mediates the protective effects of E2 against PH (Umar et al., 2011; Alencar et al., 2017). This protective effect is mainly based on studies assaying heart and peripheral vascular system function. Reportedly, estrogen is a risk factor for idiopathic PH in women, granting a longer life expectancy compared to men, due to the cardiovascular protection, a phenomenon known as “the estrogen paradox” (Umar et al., 2012; Lahm et al., 2014).

Throughout decades of estrogen research on CVDs, several studies have demonstrated that endothelial ERα participates in E2-mediated effects against atherosclerosis in low-density lipoprotein (LDL) receptor-deficient mice (Billon-Galés et al., 2009). In hepatocytes of female mice, ERα deletion increases serum cholesterol levels and high-density lipoprotein (HDL) particle size, which finally leads to an increase in atherosclerotic lesions, indicating that hepatocyte ERα signaling is crucial for reverse cholesterol transport and protection against arterial lipid accumulation in female mice models (Zhu et al., 2017). However, although there is evidence to support the atheroprotective properties of ERβ (Billon-Galés et al., 2009), more research is still needed to conclude whether ERα and ERβ protect against atherosclerosis.

Finally, E2 may inhibit fibroblast proliferation and collagen synthesis. This observation is supported by several recent studies that have demonstrated that such effects depend mainly on ERβ activation (Iorga et al., 2016, 2017). Additionally, GPER30 exerts an antifibrotic role through the prevention of cardiac fibroblast proliferation and fibrosis both *in vitro* and *in vivo* (Mahmoodzadeh et al., 2010; Mahmoodzadeh and Dworatzek, 2019).

### 2.2 Estrogen receptors and cardiac cell function

**FIGURE 1:**
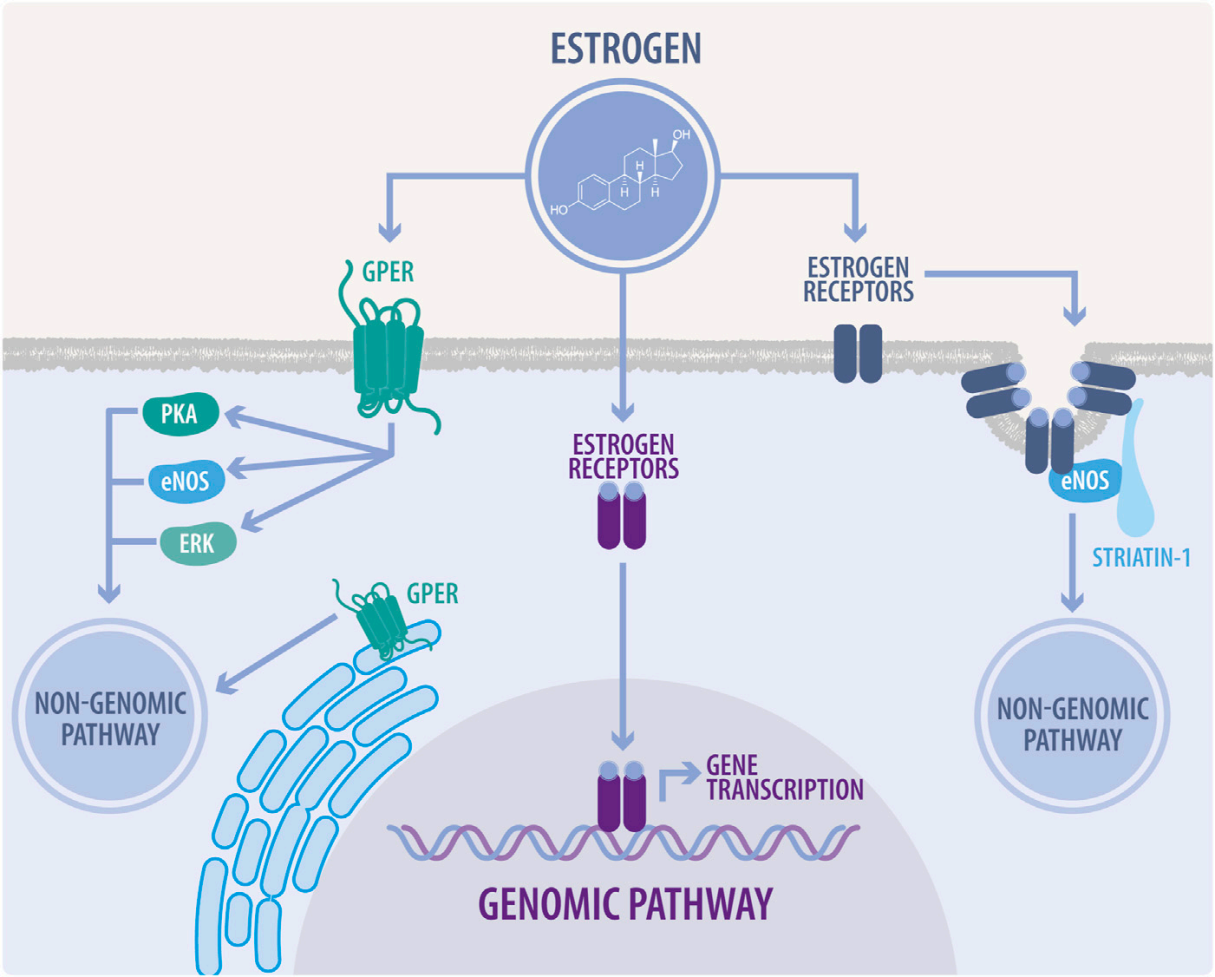
Genomic and non-genomic estrogen signaling. In the genomic pathway, estrogens diffuse across the plasma membrane and bind to estrogen receptors (ER). ERs heterodimerize and translocate to the nucleus, inducing the transcription of estrogen response element-associated genes. The non-genomic pathway is characterized by a rapid response, which modulates cellular enzyme activity and thus, directly affects cell function. In this pathway, estrogens bind to ERs associated to caveolae, mainly modulating endothelial nitric oxide synthase (eNOS) activity. They also can bind to G-protein coupled estrogen receptor (GPER) located at the plasma membrane or endoplasmic reticulum, to regulate signaling through eNOS, protein kinase A (PKA), and extracellular signal-regulated kinases (ERK), also known as mitogen-activated protein kinases (MAPK).

E2 exerts its effects through genomic and non-genomic pathways to regulate cardiovascular function (Marino et al., 2006). These effects are mediated by the classical ER: ERα, ERβ and the GPER (Figure 1); this last one has been thoroughly investigated in the last decades (Murphy, 2011).

GPER localizes to the endoplasmic reticulum and plasma membrane to exert rapid non-genomic events (Revankar et al., 2005). Several studies have shown that GPER activation protects the heart from pressure overload, ischemia, high salt diet, estrogen loss and aging in both male and female animal models. As described in mice, GPER knockout (KO) impairs glucose homeostasis and blood pressure and also produces alterations in cardiac structure and an altered systolic and diastolic function in both sexes (Sharma and Prossnitz, 2016). In the same line, analysis of cardiomyocyte DNA microarrays from GPER KO and wild type (WT) mice showed differential gene expression profiles affecting multiple transcriptional networks between male and female mice and in turn, revealed that mitochondrial genes were differentially enriched in male and female mice after cardiomyocyte-specific GPER deletion (Wang et al., 2017). In this study, the dimensions of the left ventricle in GPER KO mice were greater in males (Wang et al., 2017). These sex differences in male and female GPER KO mice may be due to the endogenous estrogen effects in females. In this regard, multiple functions of estrogens have been described in mitochondria, such as: attenuating ROS production, modulating mitochondrial ATP levels, and stabilizing the mitochondrial structural assembly (Rattanasopa et al., 2015; Wang et al., 2017). However, and despite that in recent years, several studies have specifically evaluated the effects of GPER in cardiac cells and described cardioprotection in different scenarios, its exact mechanism of action has not been determined yet.

Interestingly, a study that evaluated mice lacking the ERα showed that there were no significant cardiac differences with the WT, whereas the ERβ KO mice responded to the TAC with significant alterations in functional cardiac parameters compared to the WT. Thus, it seems that Erβ activation has a role in attenuating the hypertrophic response to pressure overload in women, which is significantly better than in their male counterparts (Skavdahl et al., 2005; Lin et al., 2009). This finding also correlates with ERβ localization in the mitochondria of cardiomyocytes in both humans and rodents, also suggesting a role for this receptor in mitochondrial integrity (Yang et al., 2004).

### 2.3 The complex relationship between estrogen and mitochondria

**FIGURE 2:**
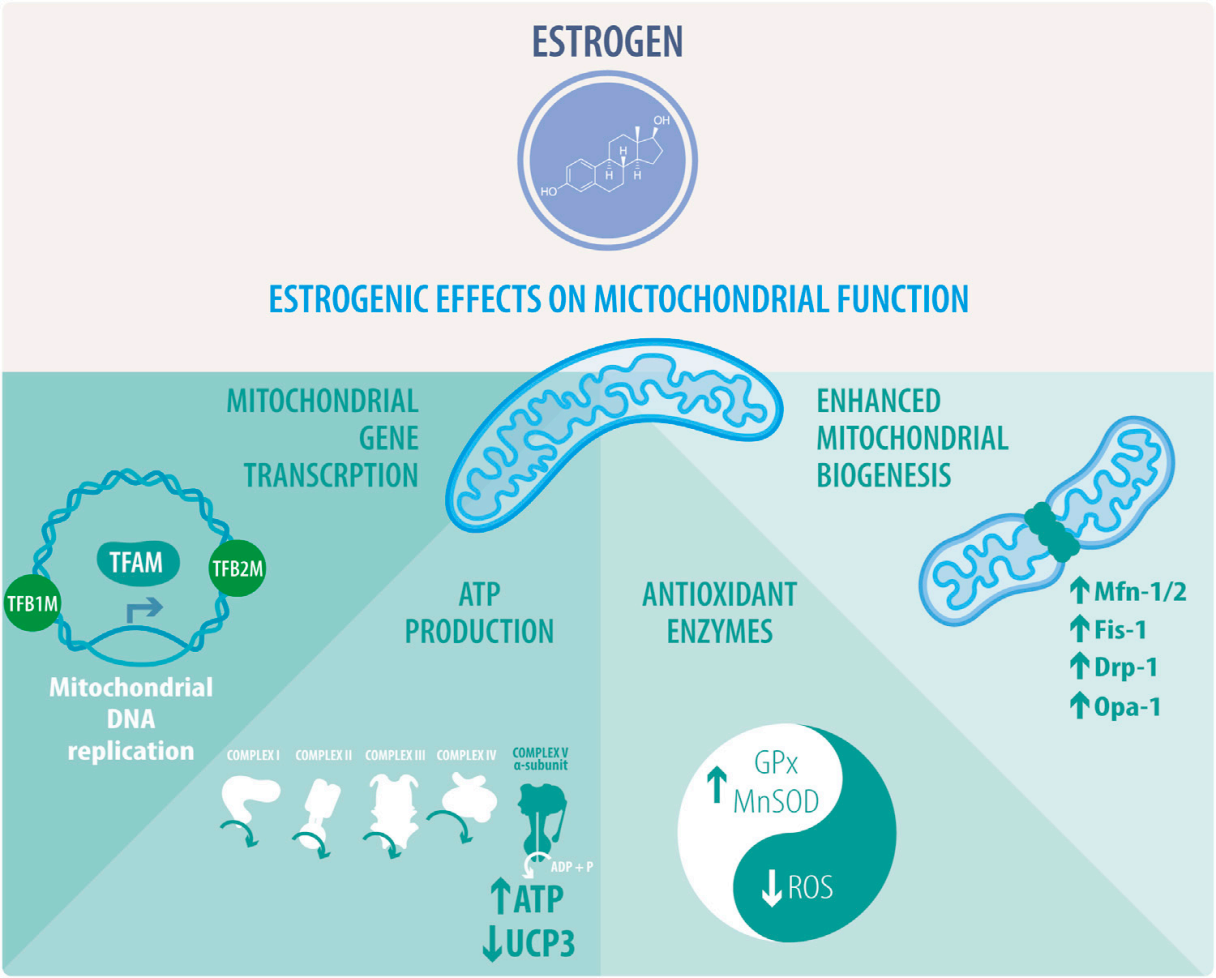
Estrogenic effects on mitochondrial function. Estrogens or activation of estrogenic pathways activate the transcription of mitochondrial genes and the replication of mitochondrial DNA thanks to the stimulation of the transcription factors TFAM, TFB1M, and TFB2M. This activity correlates with enhanced mitochondrial biogenesis and regulation of mitochondrial dynamics. Estrogens mostly favor mitochondrial fusion by increasing the expression of FIS1, MFN1/2, and OPA1. Interestingly, the rise of DRP1 activity is directly related to mitochondrial fragmentation. Estrogenic activity directly impacts ATP synthesis through oxidative phosphorylation (OXPHOS), coupled to the mitochondrial respiratory chain. This effect is related to an increase in complex V alpha subunit, subunit 1, mitochondrial respiration, and downregulation of uncoupling protein 3 (UCP3). Estrogen also preserves mitochondrial activity and integrity through the stimulation of the antioxidant enzymes glutathione peroxidase (GPx) and superoxide dismutase (SOD), leading to a decrease in reactive oxygen species (ROS).

Recent studies have suggested that mitochondria are a target of estrogens cardioprotective signaling (Figure 2), which is confirmed by the fact that many of the proposed estrogen signaling pathways converge on this organelle (Murphy, 2004; Klinge, 2017). Mitochondrial metabolism inevitably produces ROS, which in turn trigger mitochondrial dysfunction. E2 produces a decrease of ROS and increases antioxidant proteins, including superoxide dismutase 1 (SOD1), superoxide dismutase 2 (SOD2) and glutathione peroxidase (GPx) (Lynch et al., 2020). On the other hand, in the vasculature, GPER modulates ROS by decreasing NADPH oxidase 4 (NOX4), prostaglandin-endoperoxide synthase 2 (PTGS2) and GPx1, and by increasing antioxidant proteins, such as sirtuin 3 (SIRT3) and glutathione S-transferase Kappa 1 (GSTK1) (Lynch et al., 2020). Therefore, as described in several studies, females show an antioxidant difference with males that is established at the mitochondrial level, thus producing less free radicals and in turn, less cardiac oxidative damage (Borras et al., 2007; Colom et al., 2007). In this regard, some studies have reported that female mitochondria generate half the amount of hydrogen peroxide than males and have higher levels of mitochondrial reduced glutathione. However, the mechanism through which E2 performs these effects and the participation of other organelles has not yet been fully elucidated (Iorga et al., 2017). Another interesting feature that could be related to ROS modulation is the participation of E2 in the regulation of Ca^2+^ levels. Two studies have shown that OVX females exhibit mitochondria with a decreased Ca^2+^ retention capacity, which is restored after E2 administration, thus improving the normal processes of cardiac contraction and relaxation (Kravtsov et al., 2007; Wei et al., 2007; Jiao et al., 2020). Similarly, several studies have shown that regulating mitochondrial homeostasis is crucial to mitigating the disruption of different pathological processes in CVDs. Certain proteins, such as peroxisome proliferator-activated receptor coactivator 1 alpha (PGC-1α), the AMP-activated protein kinase (AMPK) and several genes involved in the electron transport chain (ETC) are regulated by sex hormones and more specifically, by estrogen signaling (Lynch et al., 2020).

The activation of GPER and ERα preserves mitochondrial function and decreases mitophagy after I/R injury through a mitochondrial permeability transition pore (MPTP)-dependent signaling and mitogen-activated protein kinase (MEK)/extracellular-signal regulated kinase (ERK) activation, thus decreasing apoptosis through the preservation of mitochondrial integrity (Feng et al., 2017; Mahmoodzadeh and Dworatzek, 2019). In this regard, estrogen administration in *in vivo* models before I/R, reduces infarct size and improves contractility (Luo et al., 2016; Mahmoodzadeh and Dworatzek, 2019). The possible mechanisms through which E2 generates these protective effects have been reviewed recently (Iorga et al., 2017). As reported, female rats are more protected against I/R injury than males in *in vivo* and isolated perfused heart models. This phenomenon could depend on mitochondria and two mitochondrial proteins. First, mitochondria from both females and E2-treated males showed increased levels of protein kinase C (PKC)-dependent phosphorylation of aldehyde dehydrogenase 2 (ALDH2), resulting in increased ALDH activity. Activation of ALDH protects the heart against ischemic damage (Chen et al., 2008). Another study also linked increased p-ALHD2 with decreased ROS production. Cardiomyocytes from female rats produced less ROS than cardiomyocytes from male rats following I/R injury (Lagranha et al., 2010). This same study demonstrated an increase in the phosphorylation of alpha-ketoglutarate dehydrogenase (αKGDH) in female hearts (Lagranha et al., 2010). αKGDH is a significant source of ROS generation, particularly under a high NADH/NAD ratio, which occurs during I/R. Permeabilized female mitochondria supplemented with αKGDH substrates and NADH decrease ROS production, suggesting that increased phosphorylation of αKGDH might reduce ROS generation (Lagranha et al., 2010). In accordance, in OVX rats, E2 deprivation decreased contents and function of respiratory complex I and IV, αKGDH, and succinate dehydrogenase; this impairment was concomitant with a decreased ROS-detoxifying enzyme activity and increased lipoperoxidation (Pavón et al., 2017). Several mechanisms of how E2 increases cardioprotection in I/R by improving mitochondrial function and increasing antioxidant activity have been recently reviewed by (Iorga et al., 2017).

Lastly, and in terms of senescence, E2 protects against cellular senescence and mitochondrial dysfunction in human umbilical vein cells, vascular smooth muscle cells (VSMC), and female C57BL/6 mice (Sasaki et al., 2021). E2 increases mitochondrial autophagy by maintaining mitochondrial function and slowing down senescence, but, interestingly, E2 does not modulate the microtubule-associated protein 1 light chain 3 (LC3); as well as the autophagy related-7 (ATG7) deficiency does not suppress mitochondrial autophagy in E2-treated cells. Moreover, the E2-mediated effects on mitochondrial autophagy were abolished by the KO of either Unc-51 like kinase-1 (Ulk1) or Ras-related protein Rab-9 (Rab9). These results suggested that E2-mediated mitochondrial autophagy is associated with Rab9-dependent alternative autophagy. Additionally, E2 upregulates sirtuin 1 (SIRT1) and activates the liver kinase B1 (LKB1), AMPK, and Ulk1, indicating that the effect of E2 on the induction of Rab9-dependent alternative autophagy is mediated by the SIRT1/LKB1/AMPK/Ulk1 pathway. Compared with the sham-operated mice, OVX mice showed reduced mitochondrial autophagy and accelerated mitochondrial dysfunction and arterial senescence, all of which were successfully rescued by E2 (Sasaki et al., 2021).

## 3 Mitochondria and their role in cardiovascular diseases

The mitochondrion is a double membrane, semiautonomous, dynamic, and densely packed organelle with a bacterial ancestry and endosymbiotic origin (Vafai and Mootha, 2012). It sustains cell life by converting carbonated skeletons to ATP, CO_2_, and H_2_O, generating oxidative stress and heat. Mitochondria are the principal energy source in different tissues and allow proper functionality of organs, especially the ones with high energy demands, such as the heart (Sun and Finkel, 2015). Mitochondria produce ATP via oxidative phosphorylation (OXPHOS), the citric cycle and β-oxidation and are the primary cellular ROS source, participating in the handling of intracellular Ca^+2^ levels and integrating survival and death signals. This organelle adapts to nutritional, oxygen and ROS conditions to maintain its function and integrity (Vásquez-Trincado et al., 2016). Interestingly, they represent 30% of the heart in volume. In cardiomyocytes, the main functional unit of the heart, mitochondria have two different populations, interfibrillar and subsarcolemmal, which are electrically coupled to each other in electrical conduction networks (Sun and Finkel, 2015).

Mitochondrial function is regulated by the formation of networks via the interaction of the outer (OMM) and inner (IMM) mitochondrial membranes of two mitochondrion, which can enhance the energetic activity of the mitochondrial network. This network then can transfer signaling molecules, lipids and Ca^+2^ within the endoplasmic reticulum at sites called mitochondria-associated membranes or MAMs (López-Crisosto et al., 2015). Mitochondrial network dynamics depend in a delicate balance between fission and fusion. In this regard, whereas mitochondrial fusion is regulated by the dynamin-related GTPases, termed Mitofusins (MFN1 and MFN2) and the optical atrophy protein 1 (OPA1), mitochondrial fission is regulated by mitochondrial fission 1 protein (FIS1) and the dynamin-related protein1 (DRP1). Perturbations of this complex interplay, mainly by an increase in fission, are closely related to CVD phenotypes (Amchenkova et al., 1988; Vásquez-Trincado et al., 2016), mainly due to increased ROS and limited energy production, which leads to apoptotic signaling and thus, mitochondrial and cardiac tissue damage (Forte et al., 2021).

In brief, mitochondrial quality maintenance is fundamental to preserving the energetic mitochondrial network and cellular homeostasis. Mitochondrial biogenesis, mitophagy, fusion, fission, and protein turnover are the processes behind this complex control, all of which will be briefly discussed below.

### 3.1 Mitochondrial dynamics in cardiovascular diseases

Mitophagy, and mitochondrial fusion/fission are coordinated to maintain energetic and cellular homeostasis. Dysregulation of any of these functions results in the accumulation of damaged mitochondria. Excessive mitochondrial fission and mitophagy compromise cell metabolic capacity (Twig and Shirihai, 2011; Morales et al., 2020). In general terms, mitochondrial fusion is linked to the removal of damaged mitochondria via autophagosomes, while fission is a requirement for mitochondrial DNA (mtDNA) distribution during cell division (Ong and Hausenloy, 2010; Vásquez-Trincado et al., 2016; Morales et al., 2020).

#### 3.1.1 Mitochondrial fusion

Mitochondrial fusion requires the coordinated action of the MFN1 and MFN2 proteins to interact with their homologues, which are located in the outer mitochondrial membrane of adjacent organelles, fusing them through a mechanism that requires GTP (Ong and Hausenloy, 2010; Vásquez-Trincado et al., 2016). On the other side, the OPA1 protein is involved in the fusion of the inner mitochondrial membranes and the remodeling of mitochondrial cristae. Moreover, OPA1 preserves the integrity and function of the internal mitochondrial membrane in response to energy damage or mitochondrial stress. In this regard, OPA1 activity depends on specific proteolytic cleavages mediated by m-AAA Protease 1 (OMA1), YME1 like 1 ATPase (YME1L1), presenilins-associated rhomboid-like protein (PARL), paraplegin and AFG3-like AAA ATPase 1 (AFG3L1) proteases (Morales et al., 2020; Forte et al., 2021).

A decrease in the functionality of the fusion machinery leads to a reduction in mitochondrial fusion, which is directly linked to CVDs. More specifically, a lower MFN2 expression is directly related to hypertension, CH and a failing heart (Vásquez-Trincado et al., 2016; Forte et al., 2021). MFN2 is also downregulated in rat models of myocardial infarction (MI), transverse aortic banding and spontaneously hypertensive rats (Fang et al., 2007). In adult cardiomyocytes, elimination of MFN1 and MFN2 induce mitochondrial dysfunction and fragmentation, leading to CH and cardiomyopathy (Chen et al., 2011; Song et al., 2015b). Specifically, among the two, MFN2 seems to be more important for mitochondrial homeostasis, since its elimination leads to early CH and cardiomyopathy (Papanicolaou et al., 2011; Chen and Dorn, 2013). On the other side, upregulation of MFN2 attenuates the CH induced by angiotensin II (Yu et al., 2011), and complementarily; in diabetes and obesity, which are conditions correlated with an increased risk of CVDs, MFN2 expression is downregulated, and can be recovered with weight loss and exercise (Bach et al., 2005; Cartoni et al., 2005).

On the other hand, specific deletion of OPA1 in mice did not greatly affect cardiac homeostasis but induced the opening of mitochondrial permeability transition pores (mPTP) (Piquereau et al., 2012). In fact, cardiac deletion of YME1L1 resulted in OMA1 activation, promoting mitochondrial fragmentation, which leads to dilated cardiomyopathy and heart failure (Wai et al., 2015). OPA1 activity largely depends on post-translational modifications (PTM); more specifically, its hyperacetylation is associated with reduced activity. In fact, cardiac stress triggers OPA1 hyperacetylation, which can be reversed by the deacetylase SIRT3, which binds directly to OPA1, promoting mitochondrial function and a substantial connection of the dynamic network (Samant et al., 2014).

#### 3.1.2 The fission mechanism

Mitochondrial fission requires the translocation of DRP1 to the mitochondria from the cytosol, promoted by PTMs, including dephosphorylation and sumoylation. This translocation is facilitated by FIS1, the mitochondrial division protein 1 (MDV1), and the mitochondrial fission factor (MFF), which are found in the OMM and act as adapter proteins. The interaction of DRP1 with these adapter proteins allows its oligomerization in a GTP-dependent process, generating a constriction ring that physically separates the mitochondrial membranes (Vásquez-Trincado et al., 2016; Morales et al., 2020; Forte et al., 2021).

DRP1 has emerged as a critical target in mitochondrial fission and cardiac research. Its cardiac-specific elimination in mice leads to a prematurely lethal phenotype associated with defective mitochondrial respiration and incomplete and flawed elimination of ubiquitinated proteins (Kageyama et al., 2014; Ishihara et al., 2015). In adult cardiomyocytes, mitophagy overactivation via Parkin upregulation induced by DRP1 elimination develops lethal cardiomyopathy (Song et al., 2015a). DRP1 inhibition protects from cardiac I/R injury and MI by decreasing mitochondrial metabolism and fragmentation (Ong et al., 2010; Disatnik et al., 2013; Zepeda et al., 2014). DRP1 deletion in adult mice leads to death in 13 weeks due to dilated cardiomyopathy with damaged mitochondria, decreased autophagy and increased cell death (Ikeda et al., 2014; Song et al., 2015b). In the case of MFF, its ablation in mouse models is lethal within 3 months. These mice show impaired mitochondrial function and increased mitophagy, although this lethal phenotype is reversed by a concomitant MFN1 deletion (Chen H. et al., 2015).

#### 3.1.3 Mitophagy

Mitophagy is the process of eliminating irreversibly damaged or dysfunctional mitochondria, targeting them to the autophagosome (Song et al., 2014). This process requires a coordinated upregulation of the mitochondrial fission machinery to precisely removing damaged mitochondrial portions. Mitophagy can occur through two mechanisms: parkin-dependent or parkin-independent (Morales et al., 2020; Forte et al., 2021). Parkin-mediated mitophagy involves PTEN-induced putative kinase 1 (PINK1) mediated-recruitment of Parkin to the OMM. PINK1 phosphorylates MFN2; then Parkin recognizes MFN2 and localizes to the mitochondria, which is an essential signal for mitophagy to start (Chen and Dorn, 2013; Xiong et al., 2019). Later, Parkin also ubiquitinates different proteins to promote their interaction with the rest of the mitophagy adaptors. One of these proteins is p62/sequestosome 1, which interacts with LC3, leading to the entrapment of the mitochondrion in the autophagosome and its subsequent digestion in the autolysosomes, after the fusion between the lysosome and the autophagosome (Forte et al., 2021). On the other hand, Parkin-independent mechanisms have also been described in some specific physiological and pathological contexts that are out of the scope of this review, wherein PINK1 phosphorylates Ub-targeted mitochondrial proteins, triggering the recruitment of the autophagy adaptors nuclear dot protein 52 kDa (NDP52) and optineurin (Lazarou et al., 2015; Morales et al., 2020). Therefore, ubiquitination of OMM proteins is a signal that is recognized by autophagy receptors, promoting the delivery of mitochondria to autophagosomal vesicles (Morales et al., 2020).

As described above, DRP1 deletion induces a decrease in mitophagy and a lethal phenotype. However, if this cardiac deletion is combined with a concomitant Parkin deletion, it results in improved cardiac remodeling and increased survival (Song et al., 2015a). In the same line, cardiomyopathy is induced by the removal of MFN2, preventing Parkin recruitment into damaged mitochondria (Chen and Dorn, 2013). Interestingly, Parkin-deficient mice show normal myocardial function (Kubli et al., 2013), despite having disorganized mitochondrial networks and significantly smaller mitochondria in their hearts. However, these Parkin^−/−^ mice were much more sensitive to MI than WT mice. After the infarction, these mice showed reduced survival and developed larger infarcts than WT mice (Kubli et al., 2013). Similarly, Parkin knockout *Drosophila* flies exhibit an accumulation of enlarged, hollow donut mitochondria with dilated cardiomyopathy (Bhandari et al., 2014). These mitochondria were depolarized despite presenting an enhanced ROS production. However, suppressing cardiomyocyte mitochondrial fusion in this model completely prevented cardiomyopathy and corrected mitochondrial dysfunction without normalizing mitochondrial dysmorphology. These results demonstrate a central role of mitochondrial fusion in cardiomyopathy provoked by impaired mitophagy (Bhandari et al., 2014).

Pink1^−/−^ mice develop left ventricular dysfunction and evidence pathological CH as early as 2 months of age. Moreover, Pink1^−/−^ mice have greater levels of oxidative stress and impaired mitochondrial function (Billia et al., 2011). In cardiomyocytes, loss of PINK1 increases the heart’s vulnerability to I/R injury due to mitochondrial dysfunction (Siddall et al., 2013). Contrastingly, PINK1 overexpression stabilizes ETC activity, increases ATP production and mitochondrial membrane potential, and inhibits mitochondrial ROS (mROS) production, therefore ameliorating I/R mitochondrial dysfunction in H9c2 cardiomyocytes (Li Y. et al., 2017). These results closely correlate with the altered mitochondrial dynamics and increased susceptibility to MI damage observed in the Parkin-deficient models (Kubli et al., 2013), suggesting that both PINK1 and Parkin play a critical role in adapting to stress in the myocardium by promoting the removal of damaged mitochondria.

Altogether, fusion, fission, and mitophagy are closely related phenomena. Fission produces a mitochondrial population characterized by a decreased size and mitochondrial membrane potential and lower OPA1 levels, thus contributing to segregating defective mitochondria and favoring the detection and removal by mitophagy (Vásquez-Trincado et al., 2016; Forte et al., 2021).

### 3.2 E2 and the regulation of mitochondrial energetics

**FIGURE 3:**
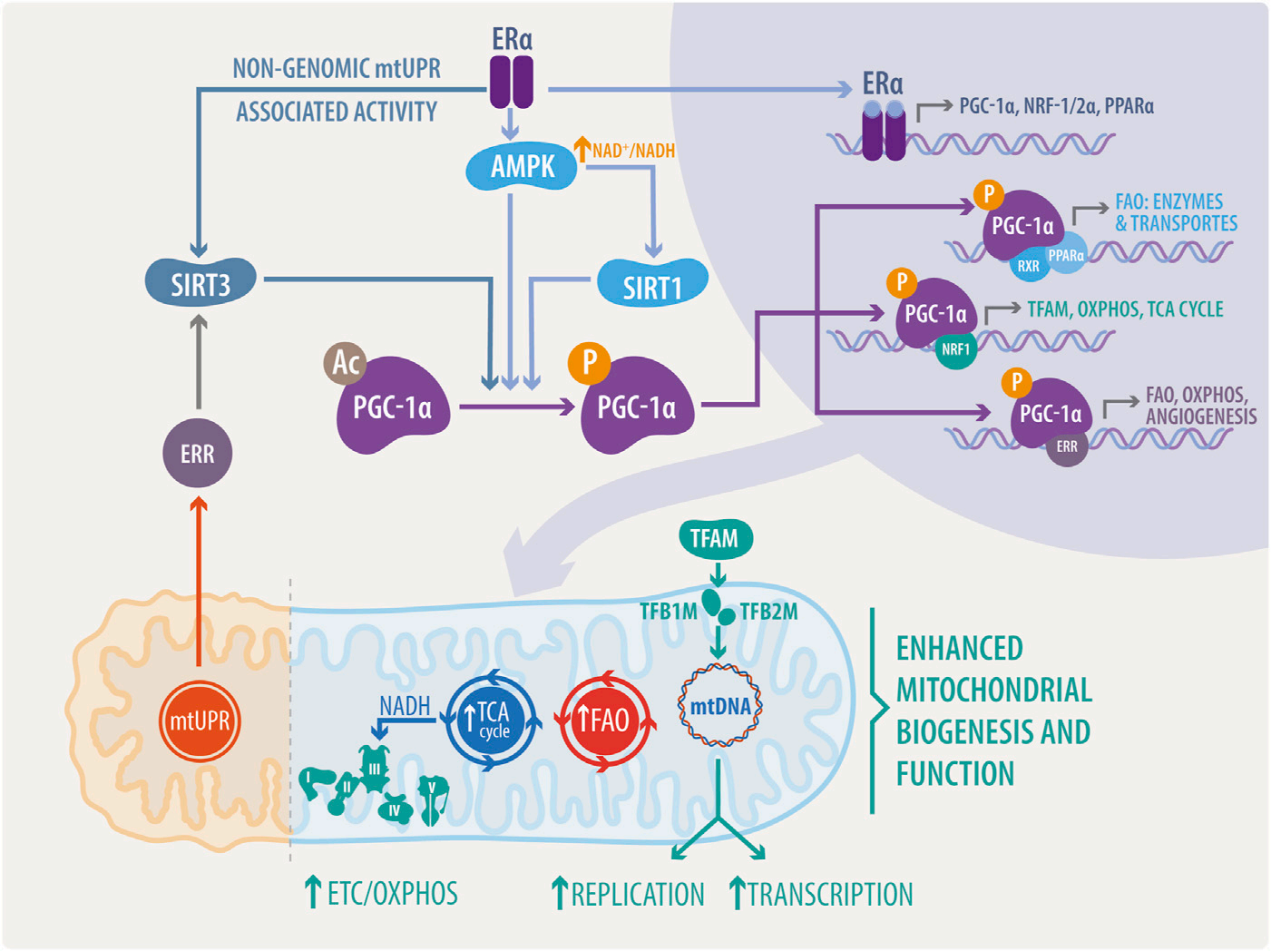
Integrated mechanisms of estrogenic and ERs activity in nucleus-mitochondria communication. PGC-1α activity, a master regulator of mitochondrial biogenesis and activity, can be regulated by SIRT1-mediated deacetylation, mtUPR associated SIRT3-mediated deacetylation via Erα/ERR, or directly through phosphorylation by adenosin monophosphate activated-kinase (AMPK). Interestingly, ERα can bind to the catalytic subunit alpha of AMPK, regulating its activity in a non-genomic manner. PGC-1α, NRF1/2α, and PPARα transcription is mediated by ERs in the nucleus. Once translated to functional proteins, these transcription factors directly regulate mitochondria, stimulating the expression of proteins, enzymes and transporters involved in mitochondrial replication and transcription, such as TFAM, OXPHOS, ETC, TCA cycle, and FAO.

Mitochondrial function is also regulated at mtDNA transcriptional level, although mtDNA is restricted to just 13 respiratory subunits. In this regard, nuclear genes play a dominant role in the biosynthesis of the respiratory chain and mtDNA expression. Thus, mitochondrial transcription is directed by the nuclear-encoded mitochondrial transcription factors (TFs) such as mitochondrial transcription factor A (TFAM; also termed mtTFA), mitochondrial transcription factor B1 (TFB1M) and B2 (TFB2M), and mitochondrial transcription termination factor (mTERF) (Figure 2). Additionally, environmental signals can induce the expression of the PGC-1 family coactivators (PGC-1α, PGC-1β, and a more distant relative the PGC-1-related coactivator [PRC]), which target specific TFs, like the nuclear respiratory factor 1 (NRF1), and 2α (NRF2α; also known as GA binding protein α: GABPα and commonly confused with NRF2) and the estrogen-related receptor (ERR) alpha (Figure 3) to regulate the expression of respiratory genes (Scarpulla, 2006; Svaguša et al., 2020; Del Campo et al., 2021).

Proteins involved in mitochondrial ETC complexes and OXPHOS are regulated by estrogens, mainly due to their genomic effects via the ERs. NRF1 expression, a gene with a functional estrogen response element (ERE) in its promoter region, TFAM, cytochrome c oxidase subunit 1, and NADH dehydrogenase subunit I are induced by estrogen in MCF-7 and H1797 cells (Mattingly et al., 2008; Azuma and Inoue, 2012). Estrogen also suppresses the expression of uncoupling protein 3 (UCP3). UCP3 is an OXPHOS uncoupling protein located in the mitochondrial inner membrane. UCP proteins uncouple ATP synthesis from the generation of the mitochondrial membrane potential in the mitochondrial respiratory chain. Therefore, ERα activity suppresses proton leakage and energy dissipation. This ERα-mediated phenomenon was confirmed by overexpressing a constitutively active receptor and treatment with ICI182,780, an ER antagonist (Nagai et al., 2016). In MCF-7 cells, estrogen increases ATP content and cyclooxygenase (COX) activity. The recently described cytochrome c oxidase subunit 7a-related polypeptide (COX7RP) functioned as a mitochondrial respiratory chain supercomplex assembly-promoting factor and was described in the context of breast and endometrial cancer. COX7RP possesses a functional ERE, and its knockdown attenuates estrogenic proliferative activity (Watanabe et al., 1998; Ikeda et al., 2019).

Additionally, E2 upregulates the transcription of MFN1, MFN2, OPA1, and DRP1 (Figure 2), induces mitochondrial fusion, and increases ATP levels; it also decreases the expression of FIS1 and OXPHOS complex proteins via ERs in MCF-7 cells (Sastre-Serra et al., 2012). Specifically, E2-activation of ERα in MCF-7 cells is required for DRP1 phosphorylation at serine (Ser) 616 via a non-genomic mechanism, increasing mitochondrial fission (Oo et al., 2018). A recent review by Lynch et al., delves into the role of estrogens in mitochondrial dynamics and biogenesis, mitochondrial-reticulum cross-communication and induction of cell death in CVDs (Lynch et al., 2020).

## 4 Estrogens as second messengers connecting the nucleus and mitochondria: Beyond the genomic and non-genomic pathway

Estrogens can bind to nuclear and membrane ERs, with different affinities for each receptor and strengths of the associated response (Watson et al., 2007). These receptors are widely expressed and differ in their structural and domain composition, which gives differential functions to the ERα and the Erβ (Pfaffl et al., 2001; Yaşar et al., 2017). In this regard, estrogens participate in different physiological functions, such as bone density, cholesterol mobilization, sexual tissues, and breast development, sexual maturation, control of inflammation, and brain function (Liang and Shang, 2013). Estrogens are also relevant in different pathological conditions. For example, in the cardiac system, a significant number of CVDs are caused by a loss of estrogenic protection, whereas pulmonary arterial hypertension is characterized by elevated plasma estrogen levels in patients (Iorga et al., 2017; Lynch et al., 2020).

Historically, the cellular action of estrogens occurs through genomic (classical) and non-genomic (non-classical) pathways (Figure 1). These pathways and the recent advances in their description will be discussed in the following sections of this review, specifically in the context of CVDs.

### 4.1 The genomic pathway

In the genomic pathway, estrogen diffuses across the plasma membrane, binding to the ERα or ERβ (Figure 1). These receptors are TFs and share a high DNA-binding domain (95%) and ligand-binding domain homology (55%). In the human and animal cardiovascular systems, these receptors are widely expressed and have demonstrated functional activity in cardiomyocytes, endothelial cells, and vascular smooth muscle cells (Ueda et al., 2019). ERs are mainly localized in the cytoplasm and nucleus of the cell; however, a fraction of ERs are localized in the plasma membrane. When the ligands bind to ERs, they change their structural conformation, releasing heat shock “chaperone” proteins (HSP) 90, 70, and 56, dimerizing and exposing binding sites for the direct interaction with chromatin at estrogen response elements (EREs). They translocate to the nucleus and, in conjunction with several other cofactors, regulate gene expression (Acconcia and Kumar, 2006). ERs have two transcriptional activation functions (AFs), as coactivators or co-repressors. AF-1, a ligand-independent region within the N-terminal region, can be phosphorylated. In particular, the ERα Ser 118, and ERβ Ser 106 and Ser 124 are critical for ligand-independent receptor activation and become phosphorylated in response to ERK/MAPK signaling. On the other hand, the C-terminal AF-2 allows ligand-dependent transcriptional activation by E2 (Acconcia and Kumar, 2006).

Based on gene expression activation analysis, ERs do not bind directly to DNA, and 35% of estrogen-regulated genes do not have EREs, thus describing an “indirect genomic pathway” or “a transcriptional cross-talk” (Fuentes and Silveyra, 2019). E2-ER complexes can modify transcription without binding directly to DNA by modulating other TFs through direct protein-protein interactions (Mendelsohn and Karas, 2005; Fuentes and Silveyra, 2019). Thus, estrogen indirect signaling influences activation or suppression of target gene expression. Specific proteins and mechanisms of this “indirect genomic pathway” have been reviewed recently by (Fuentes and Silveyra, 2019).

### 4.2 The non-genomic pathway

In the non-genomic pathway, estrogens can bind to ERα, ERβ, or the G-protein-coupled estrogen receptor (GPER) (Figure 1). This pathway modulates intracellular enzyme signaling, exerting a faster response than the genomic pathway *via* membrane-bound ERs. These effects are refractory to transcription and translation inhibitors (Puglisi et al., 2019).

The ER non-genomic signaling begins at ERs located at caveolae, activating kinases or phosphatases able to modulate cell physiology, e.g., the rapid stimulation of eNOS activity by the phosphoinositide 3-kinase (PI3K) pathway mediated by the ERα-caveolin 1 complex (Mineo and Shaul, 2012). Moreover, ERs in caveolae activate MAPK, PI3K and protein kinase B (AKT) kinases, enhancing Ser-1177 phosphorylation of eNOS. However, this is a complex process. First, ERα binds the p85 regulatory subunit of PI3K (Simoncini et al., 2000), while PI3K activation requires the proto-oncogene tyrosine-protein kinase Src (c-Src), whose SH2 domain interacts with the phosphorylated tyrosine residue (Tyr)-537 of ERα (Haynes et al., 2003; Li et al., 2007). Further, Gαi is also involved in this ERα complex at the caveolae, and the physical association of ERα with Gαi is required for eNOS activation (Wyckoff et al., 2001; Kumar et al., 2007; Ueda et al., 2019). On the other hand, striatin serves as a scaffold protein of the ERα complex at caveolae (Figure 1) (Ueda et al., 2019). In E2 responsive cells, the E2-ER interaction usually activates several signal transduction pathways, such as: Phospholipase C (PLC)/PKC; p38/MAPK; janus kinase and signal transducer and activator of transcription (JAK/STAT); p21-activated kinase 1 (PAK1); casein kinase I-g2 and sphingosine kinase (Acconcia and Marino, 2011).

As a classical G protein-coupled receptor (GPCR), the GPER can be activated by estrogen, displaying a non-genomic activity. The GPER activates the Gα subunit, and then adenylate cyclase increases 3′,5′-cyclic adenosine monophosphate (cAMP), which activates protein kinase A (PKA), with a concomitant deactivation of Raf-1 (Ciullo et al., 2001; Thomas et al., 2005). GPER also stimulates intracellular Ca^2+^ mobilization (Revankar et al., 2005; Xu et al., 2019), which is blocked by inhibition of the epidermal growth factor receptor (EGFR), thus suggesting the transactivation of the EGFR via GPER. In this pathway, the activation of GPER dissociates the G-βγ complex and activates the downstream Src-related tyrosine kinase family, as well as phosphorylation of a Shc adapter protein, enhancing matrix metalloproteinases (MMPs) expression (Filardo et al., 2000; Filardo, 2002; Revankar et al., 2005). Further in this pathway, GPER leads to the indirect activation by transactivation of MAPK/PI3K and AKT. Moreover, it also activates the c-Myc, c-fos, and c-jun TFs (McCubrey et al., 2007; Fujiwara et al., 2012; Xu et al., 2019).

### 4.3 Genomic and non-genomic activity in the heart

In cardiomyocytes, estrogen regulates the expression of connexin 43, β-myosin heavy chain, and several ion channels (Grohé et al., 1997; Stice et al., 2011). Moreover, estrogens also regulate calcineurin abundance and the activity of cGMP-dependent protein kinase (PKG) and AKT, and together with different microRNAs (miRNAs), inhibit cell hypertrophy and confer protection against apoptosis, where both nuclear and non-nuclear pathways might be involved (Patten et al., 2004; Donaldson et al., 2009; Sasaki et al., 2014; Wang et al., 2015). Furthermore, AKT activation by E2 inhibits apoptosis and activates the antioxidant machinery (Patten et al., 2004; Donaldson et al., 2009; Wang et al., 2015; Ueda et al., 2019).

In the heart, and in terms of its mitogenic activity, E2 enhances the proliferation of cardiac fibroblasts via MAPK (Wang et al., 2015; Ueda et al., 2019). In parallel, estrogenic activity stimulates endothelial cell proliferation and migration via ERα, Gi, and eNOS activation (Chambliss et al., 2010; Ueda et al., 2019). However, among 60 genes reported to be regulated by E2 in endothelial cells via ERα, 10 were also regulated by E2 in a KRR mutant model (a mice harboring a triple point mutation in ERα, preventing the binding with striatin), thus lacking non-nuclear signaling pathways (Lu et al., 2016). In contrast, E2 exerts anti-proliferative effects in VSMC. The primary mechanism involves the inhibition growth-related kinases phosphorylation, such as ERK1/2, c Jun N-terminal kinase (JNK), p38, and AKT, which are phosphorylated and activated by growth factor stimulation (Li et al., 2011; Ortmann et al., 2011). This effect is maintained via the expression and activity of several phosphatases, including mitogen-activated protein kinase phosphatase 1 (MKP1), Src homology region 2 domain-containing phosphatase 1 (SHP1), phosphatase and TENsin homolog (PTEN) and protein phosphatase 2 (PP2), which prevents the activation-mediated by phosphorylation of the growth-related kinases (Takeda-Matsubara et al., 2002; Lu et al., 2003; Yang et al., 2011). It seems that these anti-proliferative effects of E2 in VSMC occur via nuclear-independent ER signaling (Ueda et al., 2019), as evaluated in a transgenic mouse model (Disrupting Peptide Mouse; DPM), in which non-nuclear ER-mediated signaling was abolished by overexpressing a peptide representing the amino acids 176–253 of ERα, thus preventing ER from forming a signaling complex with striatin. In this mouse model, estrogen inhibition of VSMC proliferation was lost (Moens et al., 2012; Ueda et al., 2019).

Also, in terms of the vasculature, data suggest that GPER activation is protective in the vascular injury of ERα and ERβ KO mice, and that it also regulates mitochondrial function and biogenesis in OVX mice (Sbert-Roig et al., 2016; Bowling et al., 2018; Mahmoodzadeh and Dworatzek, 2019; Lynch et al., 2020). Moreover, GPER activation produces vasorelaxation through a rise of cAMP, in a dual mechanism involving endothelial NO release and inhibition of prostanoid vasoconstrictor activity (Meyer et al., 2012; Silva et al., 2021). In VSMC, GPER seems to be involved in extracellular signal-regulated kinase (ERK) phosphorylation (Haas et al., 2009) and activation of c-Fos by either the ERK or PI3K pathways (Blesson and Sahlin, 2012; Silva et al., 2021). Finally, GPER also exerts anti-inflammatory effects by downregulating interleukin (IL)-6 expression in macrophages through the suppression of nuclear factor-κB (NF-κB) activity (Okamoto et al., 2017; Silva et al., 2021).

### 4.4 Estrogens, transcriptional activity, and mitochondrial pathways

ERs upregulate the expression of PGC-1α and its downstream targets (Hsieh et al., 2005; Witt et al., 2008; Wickramasekera and Das, 2014). PGC-1α, defined as the master regulator of energy substrate metabolism and mitochondrial biogenesis, belongs to a small family of transcriptional coactivators that includes the closely related PGC-1β and a more distant relative, the PGC-1-related coactivator or PRC (Scarpulla, 2011). The effects of PGC-1a on promoting mitochondrial biogenesis and function are mediated through direct interaction and coactivation of several transcription factors, such as PPARs, ERRs, YY1, and NRF-1/2α, among others (Huss and Kelly, 2005; Scarpulla, 2011; Scarpulla et al., 2012). This explains how PGC-1α signaling is diversified into several metabolic pathways. Therefore, PGC-1α primary target genes depend on which transcription factors PGC-1α interacts with. For example, some PPARs, namely PPARδ, stimulate the expression of enzymes involved in mitochondrial fatty acid oxidation (FAO), especially in tissues and organs that require a high energy input, like the heart and skeletal muscle (Cheng et al., 2004; Wang et al., 2004; Burkart et al., 2007). ERRα, β, and γ also regulate nuclear genes encoding mitochondrial proteins involved in the tricarboxylic acid (TCA) cycle, OXPHOS, and FAO (Eichner and Giguère, 2011; Sakamoto et al., 2020). ERRα and ERRγ are highly expressed in the heart (Bookout et al., 2006). ERRα knockout downregulates the expression of mitochondrial oxidative metabolism genes. Contrastingly, this downregulation appears to have a compensatory mechanism via upregulation of ERRγ and PGC-1α. ERRα null hearts show a more severe heart failure and dilated hypertrophy, suggesting the requirement of ERRα in the energetic stress response (Dufour et al., 2007; Huss et al., 2007; Sakamoto et al., 2020). Deletion of the ERRγ also has a similar effect in reprogramming the ERRα and PGC-1α, but most of the ERRγ-null mice die within the first 7 days of life due to heart failure (Dufour et al., 2007; Fan and Evans, 2015). Hence, PGC-1α interaction with these nuclear receptors promotes mitochondrial oxidative metabolism.

ERRs are estrogen-related receptors lacking the ligand union domain. This suggests a probable estrogenic effect by direct interaction of estrogens and ERs in the transcription of ERRs and related genes, or non-genomic signaling *via* PTM in ERRs. In addition, PGC-1α promotes mitochondrial biogenesis by stimulating the expression of NRF-1/2; and directly coactivating NRF-1 on its target gene promoters (Wu et al., 1999; Gleyzer et al., 2005). Interestingly, E2 also promotes the expression of NRFs through ERα; this is mediated by the presence of an ERE in the promoter of the NRFs, which can bind both ERα and ERβ in an estrogen-dependent manner (Mattingly et al., 2008). This creates a feed-forward loop in which ER regulates PGC-1α expression, and finally, both elements regulate NRF-1 transcription, as shown in Figure 3.

NRF-1/2α controls the expression of all cytochrome c nuclear genes, the vast majority of nuclear-encoded subunits involved in OXPHOS, and proteins implicated in mtDNA replication, transcription, and translation (Kelly and Scarpulla, 2004; Dhar et al., 2008). NRF-1/2α promotes the expression of three key factors involved in mtDNA transcription, TFAM, TFB1M, and TFB2M. Once synthesized, this specific machinery translocates into the mitochondria to promote the expression of mitochondrial genes, such as the ETC proteins (Virbasius and Scarpulla, 1994; Kelly and Scarpulla, 2004; Gleyzer et al., 2005). Hence, NRF1/2α promotes mitochondrial biosynthesis by inducing TFAM, resulting in both mtRNA transcription and mtDNA replication.

Of note, several of the previously mentioned effectors have been described in the cardiovascular system in both physiological and pathological contexts. Therefore, it is not surprising that specific cardiac deletions within the PCG-1α signaling pathway can impair cardiovascular functions, and that overexpression of its components can ameliorate phenotypic dysfunction. For example, a cardiac-specific KO (cKO) of PGC-1α/β in postnatal mice caused mitochondrial fragmentation and altered expression of mitochondrial fusion (MFN1, OPA1) and fission (DRP1, FIS1) genes, and a decrease in mitochondrial respiration, finally culminating in lethality due to cardiomyopathy (Martin et al., 2014). A longevity study showed that cardiac-specific PGC-1α overexpression enhanced mitochondrial function and cardiac contractility, but accelerated cardiac aging and significantly shortened life span in 12-month-old mice because of increased mitochondrial damage and ROS (Zhu et al., 2019). Thus, maintaining adequate levels of PGC-1α is crucial for sustaining cardiometabolic homeostasis (Russell et al., 2004).

On the opposite side, PGC-1α also participates in pathological remodeling and dysfunction, as recently reviewed by (Oka et al., 2020). Most *in vivo* models of heart failure have shown downregulation of PGC-1α (Arany et al., 2006; Watanabe et al., 2014; Piquereau et al., 2017). However, some studies have not found significant changes (Oka et al., 2011; Bhat et al., 2019). Furthermore, global PGC-1α KO mice have also shown discrepancies. One study showed a normal cardiac and mitochondrial function at baseline conditions, with a mild increase in fetal gene markers (ANP, BNP, and β-MHC), and a pronounced decrease in TFAM (Arany et al., 2005, 2006). In comparison, another study indicated that mice exhibited systolic dysfunction at baseline (Leone et al., 2005). Despite this phenotypic disparity in both studies, PGC-1α KO mice exhibited a worsened response to hemodynamic stress (increased heart weight and decreased cardiac function), compared to control mice (Leone et al., 2005; Arany et al., 2006). More consistent results were reported with a cKO of PGC-1α achieved by three independent groups who implemented the same methodology to achieve the cKO. Two of them observed a mild cardiac dysfunction in cKO-PGC-1α mice at baseline conditions (Bhat et al., 2019; Kärkkäinen et al., 2019), and the other reported normal cardiac function, with females showing dilated cardiomyopathy (Patten et al., 2012). Hence, the cKO, rather than the general PGC-1α KO mice, is more prone to develop heart failure. More importantly, studies aimed at sustaining PGC-1α expression levels during pressure overload failed to report protective effects on contractile function (Karamanlidis et al., 2014; Pereira et al., 2014; Zhu et al., 2019).

Despite the unsolved questions regarding the beneficial role of PGC-1α, the modulation of TFAM has shown interesting results in heart failure models. For example, transgenic overexpression of TFAM protects mice from left ventricular remodeling and ameliorates the decrease in mtDNA copy number and mitochondrial complex enzyme activities (ETC complexes I, III, and IV) in post-MI hearts (Ikeuchi et al., 2005). More importantly, transgenic mice exhibited a higher survival rate (4 weeks) than WT, accompanied by decreased left ventricular dilatation, cardiomyocyte hypertrophy/apoptosis, and interstitial fibrosis (Ikeuchi et al., 2005). Of note, these results are consistent with similar studies of independent groups (Ikeda et al., 2015; Kunkel et al., 2019). In addition, TFAM overexpression in overload-induced heart failure models ameliorates mitochondrial ROS, decreases the expression and activity of the metalloproteinases MMP-2 and MMP9, and upregulates the expression of sarcoplasmic/endoplasmic reticulum Ca^2+^ ATPase 2a (SERCA2a) (Kunkel et al., 2019). Consistently, embryonic cKO of TFAM induced mitochondrial dysfunction and ROS production, ultimately resulting in lethal cardiomyopathy.

NRF-1 has been less described in the CVDs studies than TFAM or PGC-1α. NRF-1 expression initially increases during adaptative CH, and decreases in hypertrophic cardiomyopathy models and end-stage heart failure (Pisano et al., 2016; Nomura et al., 2018). NRF-1 regulates miR-4458 transcription in H9c2 myocytes. Interestingly, angiotensin II increases miR-4458 (and NRF-1), which in turn promotes TFAM expression by liberating TFAM mRNA from tristetraprolin (TTP), a protein involved in post-transcriptional mRNA degradation through poly-A tail removal (Yang et al., 2020). Thus, NRF-1 promotes TFAM expression through a novel mechanism mediated by miR-4458. Additionally, antioxidant supplementation with alpha-lipoic acid (α-LA) protects mice from TAC-induced left ventricular hypertrophy through the upregulation of FUN14 domain-containing protein 1 (FUNDC1), a mitochondrial membrane receptor that promotes mitophagy. Moreover, α-LA also restores ALDH2 activity, which in turn regulates FUNDC1 increase through NRF1-dependent transcription (Li et al., 2020).

In summary, we have reviewed the transcriptional cascades induced by estrogen through genomic pathways dependent on the ER. There are plenty of unanswered questions regarding PGC-1α-NRF1/2 α -TFAM axis function and regulation in failing hearts. Therefore, more studies are needed to define whether PGC-1α or TFAM are suitable therapeutic targets in CVDs.

### 4.5 Epigenetic regulation and the nucleus to mitochondria communication

**FIGURE 4:**
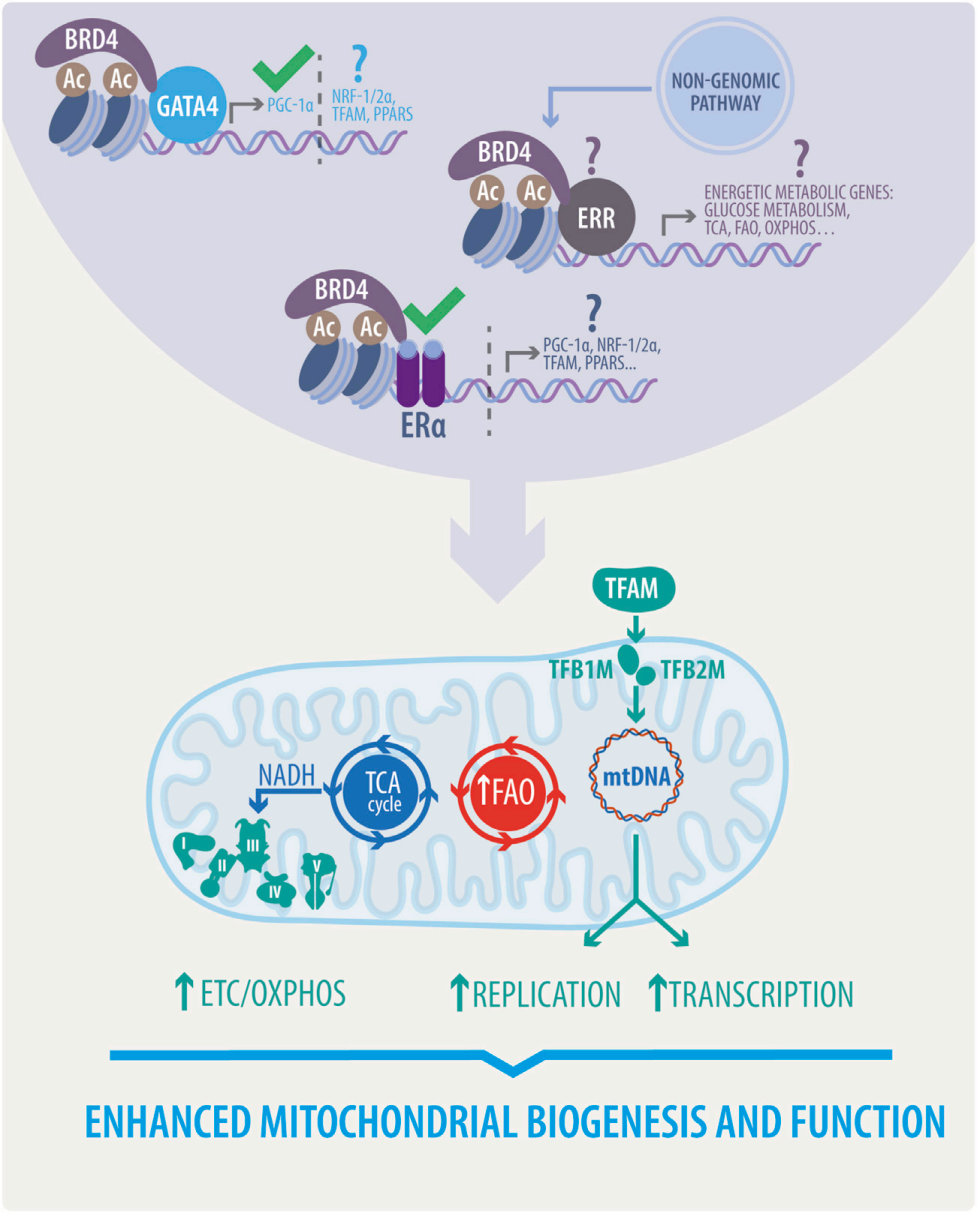
Proposed estrogenic bridge in nucleus-mitochondria communication. Estrogenic activity enhances mitochondrial biogenesis and function via upregulation of the TCA cycle, FAO, and ETC/OXPHOS activity. It also stimulates mitochondrial replication and transcription. This activity, mainly due to the function of transcription factors such as PGC1-α, NRF1/2 α, TFAM, and PPARs is initiated in the nucleus through the activation of different transcriptional complexes induced by estrogens, thereby highlighting the proposed role of BRD4 associated with GATA4, ERs or ERRs. This novel proposed mechanism would directly govern all the beneficial effects of estrogens in mitochondria. Question marks indicate effects to be yet demonstrated, and approval marks indicate what has been described.

Epigenetic regulation is also crucial in estrogen-dependent communication between the nucleus and mitochondria (Kim et al., 2016; Garbern and Lee, 2021). Bromodomain-containing protein 4 (BRD4), the most studied Bromodomain and Extraterminal (BET) family member of acetyl-lysine reader proteins, has become a highly pursued target in cancer and several CVDs (Lovén et al., 2013; Gillette and Hill, 2015; Lin and Du, 2020). Briefly, BRD4 recognizes and directly associates with acetylated chromatin at active enhancers and promoters, where it cooperates with a wide variety of TFs, to promote transcription elongation (Itzen et al., 2014; Stratton et al., 2016). Furthermore, as described in Figure 4, BRD4 can act as a coregulator of ER-dependent gene transcription (Nagarajan et al., 2014; Murakami et al., 2019). JQ1 (a pan-BET inhibitor) simultaneously inhibits E2-dependent gene transcription and proliferation in ER-positive breast cancer cells (Nagarajan et al., 2014).

Initial reports in the CVDs context suggested that BET family proteins and BRD4 were crucial participants in pathological cardiac remodeling and heart failure pathogenesis. For example, JQ1 was found to block agonist-induced *in vitro* CH, and to prevent the development of pressure overload-induced CH in mice (Anand et al., 2013; Spiltoir et al., 2013). Moreover, siRNA knockdown of BRD4 in neonatal cardiomyocytes inhibited the hypertrophic response triggered by phenylephrine and diminished the expression of fetal gene program markers (ANP and BNP) associated with CH (Anand et al., 2013). In addition to these effects, recent transcriptomics studies suggest that inhibition of BET proteins involves non-cardiomyocyte pathways, such as fibrosis and inflammation (Zhu et al., 2020). As reported, JQ1 administration in mice subjected to TAC or MI blocks the transactivation of a pathological gene program preferentially enriched in NF-κB and transforming growth factor β (TGF-β) signaling networks (Duan et al., 2017; Antolic et al., 2020). Similar results were reported *in vitro* with human-induced pluripotent stem cell-derived-cardiomyocytes (iPSC-CM) treated with endothelin-1 (Duan et al., 2017). Increased levels of BRD4 were also reported in a mouse model of high-fat diet (HFD)-induced diabetic cardiomyopathy. Upregulation of BRD4 blocked mitophagy through PINK1/Parkin modulation, resulting in the accumulation of damaged mitochondria and subsequent impairment of cardiac structure and function. BDR4 represses PINK1 transcription, and further administration of JQ1 restored mitochondrial function via PINK1/Parkin-mediated mitophagy (Mu et al., 2020).

Despite the confirmed therapeutic potential, the precise mechanism through which BET protein inhibition ameliorates cardiac remodeling has yet to be defined. Moreover, the mentioned paradigm of BET inhibition has been the subject of debate since most recent studies have suggested that, unlike pharmacological inhibition, genetic loss of BRD4 triggers a progressive decline in cardiac function (Kim et al., 2020; Padmanabhan et al., 2020). As demonstrated by two independent groups (Kim et al., 2020; Padmanabhan et al., 2020), cardiomyocyte-specific deletion of BRD4 in developing and adult hearts leads to acute deterioration of cardiac contractile function and culminates in lethality. Consistently, transcriptional profiling by RNA sequencing (RNA-Seq) experiments revealed that mitochondrial bioenergetics gene signature was preferentially downregulated in Brd4-cKO, characterized by a decrease of the master regulators PGC-1α/β and disrupted mitochondria show mild swelling (Padmanabhan et al., 2020). Functional analysis of isolated mitochondria exhibited a reduction of the electron transport chain and TCA cycle activity and protein expression (Kim et al., 2020). Moreover, genome-wide occupancy data showed that BRD4 preferentially co-localizes with GATA binding protein 4 (GATA4, a widely described TF in CVDs) at regulatory regions controlling mitochondrial bioenergetics. Furthermore, BRD4 and GATA4 directly interact in cardiomyocytes, forming an endogenous complex capable of commanding mitochondrial homeostasis through PGC-1α expression (Padmanabhan et al., 2020).

Considering the detrimental outcomes of BRD4 KO on cardiac function, JQ1 protective effects could be exerted by other BET members expressed in the heart, such as bromodomain-containing proteins 2 (BRD2), and 3 (BRD3). However, genetic approaches to delete those proteins are needed to assign specific roles. Finally, whether ERs are involved in BRD4-dependent mitochondrial cardiac homeostasis is yet to be defined. As mentioned before, ER-E2 can induce PGC-1α expression; in addition, BRD4 participates in ER-dependent transcription and further regulates PGC-1α through GATA4 (Nagarajan et al., 2014; Padmanabhan et al., 2020). These facts raise several questions about whether ERs control mitochondrial function through BRD4 coactivation, and the same query is valid for other TFs mentioned earlier. As reported, BRD4 interacts with several TFs involved in mitochondrial gene networks and cardiac physiology (Kim et al., 2020; Padmanabhan et al., 2020).

## 5 Non-classical pathways and mitochondrial to nucleus communication

So far, we have discussed how estrogens control mitochondrial function through nucleus anterograde signals and non-genomic mechanisms. However, mitochondria themselves can generate a broad range of retrograde signals towards the nucleus in order to activate the expression of nuclear-encoded genes implicated in metabolic reprogramming to protect against mitochondrial dysfunction and metabolic stress (Kotiadis et al., 2014; Quirós et al., 2016; Yong and Tang, 2018). Retrograde signals originating in the mitochondria are commonly classified as energetic deprivation and imbalance responses, ROS stress responses, Ca^2+^-dependent responses, and mitochondrial unfolded protein response (mtUPR)-dependent responses (Kotiadis et al., 2014; Quirós et al., 2016). Covering all the signaling pathways initiated by mitochondria is beyond the scope of this review, so we will focus on the four most important ones, with a special focus on energetic deprivation.

### 5.1 Energy deficit and decreased mitochondrial ATP production: The AMPK and mitochondria-dependent anterograde communication

Alterations in OXPHOS or the ETC directly impair mitochondrial ATP production, thus increasing the adenosine diphosphate (ADP)/ATP ratio, and directly stimulating AMPK. AMPK can in turn, activate the PGC-1α/NRF1/2/TFAM axis, which stimulates mitochondrial energy metabolism and biogenesis, as described in the previous sections (Jäger et al., 2007; Garcia-Roves et al., 2008; Preobrazenski et al., 2021). Interestingly, enhanced AMPK activity promotes PGC-1α transcription through phosphorylation of Forkhead box O3 (FOXO3) and cAMP response element-binding (CREB) proteins (Wright et al., 2007; Brenmoehl and Hoeflich, 2013; Vaughan et al., 2014). Additionally, AMPK triggers the mitochondrial quality control program, which regulates mitochondrial dynamics and stimulates mitophagy through inhibition of the mechanistic target of rapamycin complex 1 (mTORC1) signaling (direct phosphorylation of RAPTOR subunit and upstream regulator, tuberous sclerosis complex 2 [TSC2]) and by activating the ULK complex (Inoki et al., 2003; Gwinn et al., 2008; Kim et al., 2011; Reis et al., 2021). Hence, AMPK can potentially enhance the biogenesis of new mitochondria and energy production through induction of PGC-1α, and, concomitantly, promote the clearance of defective organelles.

Metabolic imbalance and energy deprivation not only involves intracellular ATP levels. It is well known that cellular NAD^+^/NADH levels are key regulators of metabolism and bioenergetics (Klinge, 2020; Maissan et al., 2021). Electrons derived from substrate catabolism are carried out by NADH and used for OXPHOS and biosynthetic reactions. These redox reactions are not only necessary for mitochondrial function and cell metabolism, but also for the modulation of cell signaling (Klinge, 2020). For example, SIRT1 is a NAD^+^-dependent deacetylase that senses energetic stress as an increase in the NAD^+^/NADH ratio (Li P. et al., 2017; Maissan et al., 2021). SIRT1 activates PGC-1α through deacetylation, which promotes PGC-1α translocation into the nucleus (Gerhart-Hines et al., 2007). Interestingly, SIRT1 can be further regulated by AMPK. AMPK enhances SIRT1 activity by increasing cellular NAD^+^ in C2C12 myocytes, thus resulting in deacetylation of downstream SIRT1 targets, including the PGC-1α, forkhead box O1 (FOXO1), and FOXO3a TFs (Cantó et al., 2009, 2010).

AMPK activity can be directly modulated by the E2 and ER activity (Figure 3). E2 targets AMPK through activation of ERα and direct binding with the α-catalytic subunit of AMPK, within the βγ-subunit-binding domain (Lipovka et al., 2015). Silencing of AMPKα2 downregulates ERRα. In the contrary, overexpression of ERRα in AMPKα2 knockout neonatal cardiac myocytes partially rescued the expression of energy metabolism-related genes (Hu et al., 2011). These data suggest a loop of estrogenic activation in metabolic reprogramming *via* the ERs-AMPK-ERRs pathway.

### 5.2 ROS-dependent responses

Approximately 90% of physiologically generated ROS are mROS. mROS are generated through aerobic metabolism as secondary products of ETC at complexes I and III. They function directly by regulating redox biology and as signaling molecules under physiological and pathologic conditions (Panth et al., 2016). Physiological increases in ROS levels induce a retrograde signal that initiates an antioxidant response program designed to activate detoxification enzymes and scavenger proteins (Tan et al., 2008; Nguyen et al., 2009; Lu et al., 2012; Yong and Tang, 2018). This activation is mediated by the binding of TFs to antioxidant response elements. For example, ROS impairs the kelch-like ECH-associated protein 1 (KEAP1)-mediated proteasomal degradation of nuclear factor erythroid 2-related factor 2 (NFE2L2; also known as NRF2, but not to be confused with NRF2α mentioned above), thus facilitating the translocation of NFE2L2 to the nucleus and the subsequent activation of the antioxidant program (Nguyen et al., 2009).

ROS can also induce mitochondrial biogenesis and metabolic reprogramming through AMPK-mediated PGC-1α activation and further upregulation of PGC1α expression and protein levels (Ren and Shen, 2019). Additionally, in order to regulate mitochondrial biogenesis, PGC1α can also mediate antioxidant responses (Klinge, 2020). PGC1-α coactivates the expression of SIRT3 through ERRα, which binds to the SIRT3 proximal promoter (Giralt et al., 2011; Klinge, 2020). SIRT3 localizes within mitochondria to modulate several key enzymatic activities (acyl-CoA dehydrogenase, succinate dehydrogenase, and isocitrate dehydrogenase 2, among others) and optimize metabolic function (Verdin et al., 2010). Importantly, SIRT3 is also required for the PGC-1α-mediated induction of ROS-detoxifying machinery components, such as SOD2 and GPx1, and components of the respiratory chain, such as ATP synthase 5c and cytochrome c (Kong et al., 2010).

### 5.3 Ca^2+^-dependent responses

Mitochondria are the second most important Ca^2+^ storage within cells, and a key regulator of intracellular Ca^2+^ levels (Rizzuto et al., 2012; Williams et al., 2013). Different mitochondrial stressors, such as mtDNA or leakage, disruption of ETC complexes and OXPHOS, trigger the loss of the mitochondrial membrane potential and subsequent release of Ca^2+^ into the cytoplasm (Contreras et al., 2010; Quirós et al., 2016; Srinivasan et al., 2016). Increased levels of free cytosolic Ca^2+^ can induce a complex transcriptional cascade involving several TFs and signaling effectors. For example, Ca^2+^ activates calcineurin, a phosphatase that activates the nuclear factor of activated T cells (NFAT) and NF-κB p105, which is also directly activated by mROS (Quirós et al., 2016; Chowdhury et al., 2020). NFAT and NFκB have been widely described as cardiac TF involved in pathological cardiac remodeling (Tham et al., 2015; Fiordelisi et al., 2019). Dephosphorylated NFAT translocates towards the nucleus, interacting with GATA4 and the myocyte enhancer factor-2 (MEF2) (Suzuki et al., 2002; Tham et al., 2015; Stansfield et al., 2014). Activated NFAT promotes the transcription of hypertrophy-associated genes (also known as the fetal gene program), including α-actin, endothelin-1, ANP, and β-MHC (Heineke and Molkentin, 2006; Luo et al., 2021). Furthermore, NFAT inhibition (throughout FOXO overexpression or knockdown) ameliorates hypertrophy *in vitro* (Ni et al., 2006; Li et al., 2018; Coleman et al., 2021). Interestingly, NFAT and NFκB directly interact to command and coordinate two independent signaling pathways that promote CH (Liu et al., 2012).

In addition, elevated intracellular Ca^2+^ levels also activate a variety of Ca^2+^-dependent kinases, namely Ca^2+^/calmodulin-dependent protein kinase type IV (CAMKIV), PKC, JNK, p38 MAPK, among others (Quirós et al., 2016; Stansfield et al., 2014). These, in turn, activate other TFs, such as early growth response protein 1 (EGR1), CREB, and CEBP homologous protein (CHOP), among others (Arnould et al., 2002; Woods et al., 2005; Heineke and Molkentin, 2006). Activation of these TFs and their downstream targets involves mitochondrial adaptation and leads to several responses regarding Ca^2+^ metabolism, insulin signaling, and cell proliferation. In fact, these TFs have also been reported in both physiological and pathological CH. In this regard, calcineurin directly regulates ERα stability and activity in breast cancer and estrogen upregulates calcineurin expression via overexpression of ER in systemic lupus erythematosus (Rider et al., 2000; Lin et al., 2011; Masaki et al., 2021). Similarly, in the heart, estrogen inhibits isoproterenol-induced CH via suppression of Ca^2+^-calcineurin signaling, preventing NFATc3 translocation (Tsai et al., 2017). Moreover, in a rat vascular responsiveness model, estrogen increases vascular reactivity via activation of GPER-Rho kinase and PKC pathway activation, but not exclusively due to the genomic and non-genomic responses (Li et al., 2014). This estrogenic protection mechanism also appears in I/R models via a mechanism dependent on PKCε and ERα (Novotny et al., 2009). Interestingly, it has been shown that mitochondrial calcium uniporter (MCU), the main responsible for mitochondrial Ca^2+^ uptake, is strongly regulated by agonists and antagonists of ERs. In particular, the specific alpha-ER agonist 4,4′,4''-(4-propyl-[1H]-pyrazole-1,3,5-trial) trisphenol was the most potent activator, increasing the rate of mitochondrial Ca^2+^ uptake (Lobatón et al., 2005), thus suggesting that a nongenomic mechanism regulates MCU activity. Available literature shows that estrogen deficiency deregulates L-type Ca^2+^ channel, ryanodine receptor, SERCA and the Na^+^-Ca^2+^ exchanger, causing impaired Ca^2+^ homeostasis, thus leading to CVDs, as recently reviewed by (Jiao et al., 2020).

Considering the foregoing and the evidence for Ca^2+^ overload and calcineurin inhibition as a central hub in the protective estrogenic activity in CH (Pedram et al., 2008), a future projection in the field is the elucidation of the transcriptional program mediated by Ca^2+^-estrogen–Ca^2+^ phosphatases and the related TFs, as well as their role in the estrogenic CVDs protective programming.

### 5.4 Mitochondrial UPR-dependent responses

The mtUPR is an evolutionarily conserved mechanism activated in response to a compromised mitochondrial protein folding environment and misfolded protein accumulation (Haynes and Ron, 2010; Muñoz-Carvajal and Sanhueza, 2020). Further, mtUPR orchestrates several responses, including the antioxidant machinery, the OXPHOS functioning, mitophagy, the process of mitochondrial protein quality control, and mitochondrial biogenesis (Haynes and Ron, 2010). Consistent with the endoplasmic reticulum UPR, mtUPR initiates a nuclear anterograde signaling including the activating transcription factor 5 (ATF5) and CHOP as key TFs that enhance the transcription of several mitochondrial protective genes (including several chaperones, proteases, antioxidant enzymes, and the glycolytic machinery) that operate to restore mitochondrial protein homeostasis and ensure cell survival (Haynes and Ron, 2010; Melber and Haynes, 2018; Muñoz-Carvajal and Sanhueza, 2020). The precise molecular mechanisms and mediators of the mtUPR have been widely described in *C. elegans* (please refer to Quirós et al., 2016 and Haynes and Ron, 2010 for a more comprehensive review of this description). Comparatively, little is known about the mammalian mtUPR, especially in cardiovascular physiology (Zhou et al., 2020).

Interestingly, recent studies have begun to describe the potential cardioprotective effects of mtUPR on CVDs models. Neonatal rat cardiomyocytes treated with complex I inhibitor paraquat, or the β-adrenoreceptor agonist isoproterenol, showed an increase in mRNA levels of ATF5, CHOP, mitochondrial pre-sequence translocase-associated motor complex protein (mtDNAj), ATP-dependent Clp protease proteolytic subunit (ClpP), mitochondrial Lon protease homolog (LonP), HSP10 and HSP60. Additionally, left ventricular tissue of mice subjected to pressure overload also displayed an increase in the mtUPR effectors ATF5, ClpP, and LonP (Smyrnias et al., 2019). Furthermore, pharmacological enhancement of mtUPR improved cardiomyocyte survival, contractile function, and mitochondrial oxygen consumption (complex I and II) in mice subjected to chronic pressure overload. Consistently, mice pretreated with oligomycin or doxycycline (mtUPR inducers) displayed an enhanced functional recovery and decreased infarct size against *ex vivo* post-I/R injury. Interestingly, this protection was abolished upon ATF5 depletion, demonstrating the essential role of this TF in mediating the mtUPR cardioprotective effects (Smyrnias et al., 2019; Wang et al., 2019).

This review shows that estrogenic mechanisms are a master controller of anterograde and retrograde nucleus-mitochondria responses and regulation. However, in terms of mtUPR, this communication network has not been adequately described yet in CVDs, although we can find information on cancer mtUPR mechanisms (Jenkins et al., 2021a; 2021b) that can be used as references for CVDs. Estrogen-dependent mtUPR mechanisms in CVDs are still not described. Still, some works have shown ERα activity and sirtuins-mediated post-translational modifications (Jenkins et al., 2021b) which can be interpreted in this context, as we discuss next.

Sirtuins are a family composed of seven proteins regulating longevity, metabolism, and response to stress. The NAD-dependent deacetylase SIRT3 is specifically found in the mitochondria, where is related to proteotoxic matrix stress and directly regulated by estrogenic pathways (Papa and Germain, 2014; Germain, 2016; Zhang et al., 2020). These pathways are controlled by both estrogen-dependent and estrogen-independent ERα activation mechanisms (Jenkins et al., 2021a). In the estrogen-independent mechanisms, ERα is activated by AKT-mediated phosphorylation (Bhat-Nakshatri et al., 2008). In the estrogen-dependent mechanisms, estrogen binds and activates ERα (Ruff et al., 2000). ERα controls the cytoprotective ERα-NRF1-proteasome axis of the mtUPR (Figure 3) and enables the maintenance of the mitochondrial integrity (Papa and Germain, 2011); otherwise, SIRT3 controls SOD2 induction via FOXO3a during mtUPR in a CHOP-independent manner (Papa and Germain, 2014), enabling antioxidant activity.

We can ask ourselves, how is this pathway related to a protective estrogenic modulation of CVDs? SIRT3 is considered a new key actor in CVDs, due to its cardioprotective effects which are reflected mainly in the fact that a loss of SIRT3 expression increases the susceptibility to suffer or worsens the pathological phenotype in cardiac ischemia-reperfusion injury and coronary microvascular dysfunction, thus impairing cardiac recovery (Sun et al., 2018). Moreover, drugs that inhibit the renin-angiotensin-aldosterone system improve cardiac function and increase SIRT3 levels (Parodi-Rullan et al., 2012) in animal models of heart failure. Similarly, a trimethylamine-N-oxide (TMAO) vascular inflammation model inhibits SIRT3 expression and SOD2 activation. SIRT3 overexpression protected from TMAO injury (Chen et al., 2017). Sirt3-KO mice showed lower palmitate oxidation, lower respiratory capacity, lower ATP synthesis, and abnormal lipid accumulation, with impaired mitochondrial and contractile cardiac function (Chen T. et al., 2015; Koentges et al., 2015). Finally, low SIRT-3 levels are also correlated with a down-regulation of PGC1-α (Yu et al., 2017), suggesting the conservation of the reported PGC-1α/SIRT3 protective axis (Son et al., 2021) due to SIRT3 enhanced activity (Figure 3). Thus, all the listed findings highlight the importance of elucidate the hypothetical ERα or ERRs-SIRT3-mtUPR mitochondrial cardioprotective pathway in different CVDs as a new important axis in the cardioprotection triggered by estrogens.

## 6 Conclusion and future perspectives

The general cardiovascular protective effect of estrogens, the activation of ERs and ERRs, has been reported from preclinical models to clinical models by studying the loss of estrogenic activity and subsequent replacement. Additionally, the favorable effect on the maintenance of mitochondrial dynamics and function exerted by the estrogenic-associated activity that has been highlighted in this review, allows us to propose a central protective pathway focused in energetic and mitochondrial functionality. This is due mainly to the communication between the nucleus and mitochondria, governed by upregulation or activation of transcriptional factors, which mediate the expression of mitochondrial dynamics and energetic genes in mtDNA and nuclear DNA. This protective pathway is shown in Figure 4.

The pleiotropic action of estrogens has led to the findings of various side effects in its implementation as CVDs related therapy in men and women. Several clinical trials have been carried out using estrogens as hormone replacement therapy. These trials have analyzed the dose of estrogens, their origin, the timing of their application depending on the reproductive cycle, and the routes of administration and delivery (Shufelt and Manson, 2021; Ueda et al., 2021; Anagnostis et al., 2022). Although we have indicated the existence of sex differences in the effects of hormonal therapies associated with CVDs, this review is not focused on discussing the specific benefits or possible side effects of its pharmacological use (for a more comprehensive review in this topic, please refer to Yang and Reckelhoff, 2011; Maas, 2021 and Shufelt and Manson, 2021). Nonetheless, we have to mention the relationship between estrogen usage and the increased risk of reproductive cancers (Simin et al., 2017; D’Alonzo et al., 2019; Vinogradova et al., 2020). Under this perspective, we must highlight the efforts to implement and develop new generations of drugs, such as selective estrogen receptor modulators (SERMs). This group of molecules exerts estrogenic or anti-estrogenic effects depending on target tissue or cell type, and are currently used in the treatment of reproductive cancers (Alsina and Martín, 2013). As discussed, a supposed new pharmacological therapy should exert its specific tissue effects, modulating estrogen’s positive effects on mitochondrial to nucleus communication and restoring/keeping mitochondrial function. However, to achieve successful specific therapeutic results, it is necessary to continue researching and deciphering how to emulate the estrogenic effects in the contexts of the retrograde and anterograde communication between the nucleus and mitochondria. Therefore, it is necessary to generate specific pharmacological targets for the activation of BRD4, ERRs, AMPK, SIRTs, and PGC-1α in CVDs, thus emulating the estrogenic protection without the detrimental side effects reported for the classical drugs.

Finally, it is urgent to find the transcriptional program stimulated in conjunction by BRD4 and ERs or ERRs in CVDs to confirm this pathway as a master regulator of the E2 protective effects and to clarify whether it is directly activated by genomic pathways or indirectly via the non-genomic actions of estrogens.

## Glossary

ADP: Adenosine diphosphate
AFG3L1: AFG3-like AAA ATPase 1
AFs: Activator functions
ALDH2: Aldehyde dehydrogenase 2
AMPK: Adenosine monophosphate-activated protein kinase
ANP: Atrial natriuretic peptide
ATF5: Activating transcription factor 5
ATG7: Autophagy related gene-7
ATP: Adenosine triphosphate
BET: Bromodomain and extra-terminal
BNP: Brain natriuretic peptide
BRD4: Bromodomain-containing protein 4
Ca2+: Calcium
CAMKIV: Ca2+/calmodulin-dependent protein kinase type IV
cAMP: 3′,5′-cyclic adenosine monophosphate
CH: Cardiac hypertrophy
CHOP: CEBP homologous protein
cKO: Cardiac-specific knockout
ClpP: ATP-dependent Clp protease proteolytic subunit
COX: Cyclooxygenase
COX7RP: Cytochrome c oxidase subunit 7a-related polypeptide
CREB: cAMP response element-binding protein
c-Src: Proto-oncogene tyrosine-protein kinase Src
CVDs: Cardiovascular diseases
DPM: Disrupting peptide mouse
DRP1: Dynamin-related protein1
E2: 17 β-estradiol
EGFR: Epidermal growth factor receptor
EGR1: Early growth response protein 1
eNOS: Endothelial nitric oxide synthase
ER: Estrogen receptor
ERE: Estrogen response element
ERK: Extracellular-signal regulated kinase
ERR: Estrogen related receptor
ERα: Estrogen receptor alpha
ERβ: Estrogen receptor beta
ETC: Electron transport chain
FAO: Fatty acid oxidation
FIS1: Mitochondrial fission 1 protein
FOXO1: ForkHead box O1
FOXO3: Forkhead box O3
FUNDC1: FUN14 domain-containing protein 1
GATA4: GATA binding protein 4
GPCR: G protein-coupled receptor
GPER: G protein-coupled estrogen receptor
GPx: Glutathione peroxidase
GSTK1: Glutathione S- transferase kappa 1
GTP: Guanosine triphosphate
HFD: High-fat diet
I/R: Ischemia-reperfusion
IL: Interleukin
IMM: Inner mitochondrial membrane
iNOS: Inducible nitric oxide synthase
IPSC-CM: Induced pluripotent stem cell derived-cardiomyocytes
JAK/STAT: Janus kinase and signal transducer and activator of transcription
JNK: c-Jun N-terminal kinase
KD: Knockdown
KEAP1: Kelch-like ECH-associated protein 1
KO: Knockout
LC3: Microtubule-associated protein 1 light chain 3
LDL: Low-density lipoprotein
LKB1: Liver kinase B1
LonP: Mitochondrial Lon protease homolog
MAMs: Mitochondria associated membranes
MAPK: Mitogen-activated protein kinase
MCU: Mitochondrial Ca^2+^ uniporter
MDV1: Mitochondrial division protein 1
MEF2: Myocyte enhancer factor 2
MEK: Mitogen-activated protein kinase kinase
MFF: Mitochondrial fission factor
MFN1: Mitofusin 1
MFN2: Mitofusin 2
MI: Myocardial infarction
miRNAs: MicroRNAs
mtUPR: mitochondrial unfolded protein response
MKP1: Mitogen-activated protein kinase phosphatase 1
MPPs: Metalloproteinases
MPTP: Mitochondrial permeability transition pore
mROS: Mitochondrial ROS
mtDNA: Mitochondrial DNA
mtDNAj: Mitochondrial pre-sequence translocase-associated motor complex protein
mTERF: Mitochondrial transcription termination factor
mTORC1: Mechanistic target of rapamycin complex 1
NFAT: Nuclear factor of activated T cells
NFE2L2: Nuclear factor erythroid 2-related factor 2
NF-κB: Nuclear factor-κB
NO: Nitric oxide
NOS: Nitric oxide synthase
NOX4: NADPH oxidase 4
NPD52: Nuclear dot protein 52 kDa
NRF1: Nuclear respiratory factor 1
NRF2α: Nuclear respiratory factor 2α
OMA1: m-AAA Protease 1
OMM: Outer mitochondrial membrane
OPA1: Optic atrophy protein 1
OVX: Ovariectomized
OXPHOS: Oxidative phosphorylation system
PAK1: p21-activated kinase 1
PARL: Presenilins-associated rhomboid-like protein
PGC-1: Peroxisome proliferator-activated receptor coactivator 1
PH: Pulmonary hypertension
PI3K: Phosphoinositide 3-kinase
PINK1: PTEN-induced putative kinase 1
PKA: Protein kinase A
PKC: Protein kinase C
PKG: cGMP-dependent protein kinase
PP2: Protein phosphatase 2 (PP2)
PPARs: Peroxisome proliferator-activated receptors
PTEN: Phosphatase and TENsin homolog
PTGS2: Prostaglandin-endoperoxide synthase 2
PTMs: Post-translational modifications
RAB9: Ras-related protein Rab-9
RNA-Seq: RNA sequencing
ROS: Reactive oxygen species
Ser: Serine residue
SERCA2a: Sarcoplasmic/Endoplasmic reticulum Ca^2+^ATPase 2a
SHP1: Src homology region 2 domain-containing phosphatase 1
SIRT1: Sirtuin 1
SIRT3: Sirtuin 3
SOD1: Superoxide dismutase 1
SOD2: Superoxide dismutase 2
TAC: Transverse aortic constriction
TCA: Tricarboxylic acid
TFB1M: Mitochondrial transcription factor B1
TFB2M: Mitochondrial transcription factor B2
TFs: Transcription factors
TGF-β: Transforming growth factor β
TMAO: Trimethylamine-N-oxide
TSC2: Tuberous sclerosis complex 2
TTP: Tristetraprolin
Tyr: Tyrosine residue
UCP3: Uncoupling protein 3
ULK1: Unc-51 like kinase 1
VSMC: Vascular smooth muscle cells
WT: Wild type
YME1L1: YME1 like 1 ATPase
α-LA: α-lipoic acid
αKGDH: α-ketoglutarate dehydrogenase
β-MHC: Myosin heavy chain, cardiac muscle β-isoform
